# A snapshot of genetic and epigenetic basis of arrhythmia and heart failure

**DOI:** 10.3389/fgene.2015.00074

**Published:** 2015-03-03

**Authors:** Junjie Xiao, Joost P. G. Sluijter, Saumya Das, Yiqing Yang, Zhongming Shen

**Affiliations:** ^1^Regeneration and Ageing Lab, Experimental Center of Life Sciences and Innovative Drug Research Center, School of Life Science, Shanghai UniversityShanghai, China; ^2^Shanghai Key Laboratory of Bio-Energy Crops, School of Life Science, Shanghai UniversityShanghai, China; ^3^Division Heart and Lungs, Department of Cardiology, University Medical Center UtrechtUtrecht, Netherlands; ^4^Cardiovascular Research Institute, Beth Israel Deaconess Medical Center, Harvard Medical School, Harvard UniversityBoston, MA, USA; ^5^Department of Cardiology, Shanghai Chest Hospital, Shanghai Jiao Tong UniversityShanghai, China

**Keywords:** genetic variation, epigenetics, arrhythmia, heart failure, microRNAs

The incidence of cardiac arrhythmia and heart failure continues to rapidly increase, leading to a growing epidemic which constitutes a serious financial burden for society (Chugh et al., 2014; Melman et al., 2014). Despite the standard therapeutic strategies to treat adverse cardiac remodeling that precedes the development of arrhythmia and overt heart failure, their prevalence is still increasing and their morbidity and mortality have not been largely improved (Melman et al., 2014; Sardar et al., 2014). Continued and deeper understanding of the molecular mechanisms responsible for arrhythmia and heart failure will help identify novel effective therapies.

In the past few years, research has implicated the contribution of genetic and epigenetic factors to arrhythmia and heart failure. This *research hot topic* entitled “Genetic and epigenetic basis of arrhythmia and heart failure” provides a snapshot in this area. The 10 selected papers in this special research topic will enhance current understanding of the genetic and epigenetic basis of arrhythmia and heart failure, and also will expand our knowledge on novel interventions.

In this issue, genetic aspects of arrhythmia were described by 3 different groups. Crump and Abbott (2014) provided an update about arrhythmogenic KCNE gene variants and their roles, Nielsen et al., (2013) showed that the I_to_ regulatory subunit Klf15 was not associated with early-onset long atrial fibrillation in their cohort. Besides familial atrial fibrillation, genome wide association studies (GWAS) have identified common variants in the general population for atrial fibrillation. Yao et al. (2014) gave an overview on the paired-like homeodomain 2, which was identified as a common variant to be involved in atrial fibrillation, highlighting the potential of paired-like homeodomain 2 as a new therapeutic target.

Duygu et al. (2013) provided an outstanding overview about the genetics and epigenetics of arrhythmia and heart failure. Besides highlighting specific genetic mutations associated with heart failure, they also summarized epigenetic mechanisms including DNA methylation, ATP-dependent chromatin remodeling, histone modification and RNA-based mechanisms, highlighting the relationship among epigenetics, heart failure and arrhythmiaogenesis. Finally, they also introduced a discussion on the role of non-coding RNAs especially microRNAs (miRNAs, miRs) in heart failure and arrhythmia.

miRNAs are endogenous non-coding RNAs which post-transcriptionally regulate gene expressions (Condorelli et al., 2014). A single miRNA can target several target genes while an individual gene can be regulated by many miRNAs (Viereck et al., 2014). Therefore, miRNAs have been considered as central regulators of gene expression, which participate in many essential biological processes including cellular proliferation, differentiation, apoptosis, and cardiac metabolism (Condorelli et al., 2014; Viereck et al., 2014). Dysregulations of miRNAs have been connected to many cardiovascular diseases, including arrhythmia and heart failure (Kwekkeboom et al., 2014; Melman et al., 2014). In this special research topic, 4 opinion articles have been provided about miRNAs in arrhythmia and heart failure. Fu et al. (2014) presented the current understanding about miRNAs as novel players in atrial fibrillation, a most common form of arrhythmia that affects at least 1% persons in the general population. Changes in cardiac structure and function (termed diabetic cardiomyopathy) may play a role in the increased incidence of sudden death and heart failure in the diabetic patients. Zhou et al. (2014) summarized dysregulations of miRNAs in diabetic cardiomyopathy. Interestingly, changes of circulating miRNAs in diabetic patients have also been noted, although their associations with diabetic cardiomyopathy are unclear. Better understanding of circulating miRNAs in diabetic cardiomyopathy would help the development of biomarkers and novel therapies. Ageing is correlated with an increase in the incidence of both heart failure (particularly heart failure with preserved ejection fraction) and atrial fibrillation. Considering the rapid increase in the geriatric population, it is urgent to understand the role of miRNAs in aging. Zhuo et al. (2014) provided some insights into the dysregulation of miRNAs in aging-related heart failure. New therapeutics via targeting microRNAs are being developed based on the detailed understanding of the role of miRNAs in heart failure and arrhythmia using either antagomirs or mimics (Kwekkeboom et al., 2014). In contrast to the previous articles on the role of miRNAs in pathological heart diseases, Fu et al. (2013) dissected the role of miRNAs in physiological hypertrophy. Physiological hypertrophy, which is associated with exercise and pregnancy, is not associated with pathological processes like apoptosis and fibrosis, and unlike its counterpart (pathological hypertrophy), is not the harbinger of sudden death or heart failure. Given the known beneficial effects of exercise on cardiovascular outcomes, a better understanding of miRNAs in physiological hypertrophy may lead to novel miRNA-based therapies for heart failure.

Finally Stagnaro and Caramel (2013) proposed a “Quantum Biophysics Semeiotics” (QBS) microcirculatory theory of atherosclerosis, which could be used for diagnosis or therapies. In addition, Tao et al. (2013) provided their prospects of using Qiliqiangxin, a traditional Chinese medicine, in the treatment of heart failure potentially through epigenetic regulation of regeneration.

In conclusion, this research topic offers a detailed and updated summary about genetic and epigenetic basis of arrhythmia and heart failure, and provides a forum for discussion on ways to translate these genetic and epigenetic findings into novel therapies for arrhythmia and heart failure.

## Conflict of interest statement

The authors declare that the research was conducted in the absence of any commercial or financial relationships that could be construed as a potential conflict of interest.
